# Genetic polymorphisms of *CYP3A4* among Chinese patients with steroid-induced osteonecrosis of the femoral head

**DOI:** 10.1097/MD.0000000000005332

**Published:** 2016-11-04

**Authors:** Yuan Wang, Xiuling Li, Yaoyu Gao, Zhi Li, Lidong Yu, Qingbo Meng, Li Sun, Jianzhong Wang

**Affiliations:** a The People's Hospital of Manzhouli City, Manzhouli; b The Second Affiliated Hospital, Inner Mongolia Medical University; c Inner Mongolia Medical University, Hohhot, Inner Mongolia, China.

**Keywords:** case–control study, *CYP3A4*, gene, genotype analysis, single nucleotide polymorphism, steroid-induced osteonecrosis of femoral head

## Abstract

**Background:**

Steroid therapy has been an important reason of nontraumatic osteonecrosis of the femoral head (ONFH). Steroids are metabolized by hepatic cytochrome P4503A, a low endogenous activity of this enzyme can contribute to the pathogenesis of ONFH. The aim of this study was to investigate the associations of polymorphisms of *cytochrome P4503A4* (*CYP3A4*) gene with steroid-induced ONFH in Chinese patients.

**Methods:**

A total of 150 steroid-induced ONFH patients and 250 healthy controls were enrolled. We evaluated 5 single-nucleotide polymorphisms of the *CYP3A4* gene in this case–control study.

**Results:**

We identified rs2242480 in the *CYP3A4* gene that was potentially associated with an increased risk of steroid-induced ONFH in the allele model (*P* = 0.023; odds ratio [OR]: 1.47; 95% confidence intervals [CI]: 1.05–2.04) and in the additive model (*P* = 0.028; OR: 1.44; 95% CI: 1.04–1.99) adjusted age + gender. Furthermore, we also observed a protective effect of haplotype “TG” (*P* = 0.025; OR: 0.69; 95% CI: 0.49–0.96) and a risk effect of haplotype “CG” (*P* = 0.006; OR: 1.81; 95% CI: 1.19–2.77) of the *CYP3A4* gene adjusted age + gender.

**Conclusion:**

These findings suggested that polymorphisms of *CYP3A4* gene may be associated with susceptibility to steroid-induced ONFH.

## 1 Introduction

Osteonecrosis of the femoral head (ONFH) is osteocyte death leading to the gradual disruption of the femoral head and it is characterized for impaired differentiation of mesenchymal cells, cellular toxicity, and destruction of intravascular blood flow, ultimately resulting in bone death.^[1]^ The steroid therapy is normally specified to patients with renal transplantation, systemic lupus erythematosus, autoimmune inflammatory diseases, and nephrotic syndrome have been a central reason of nontraumatic ONFH.^[2,3]^ The prevalence of ONFH is investigated to be annually 15,000 to 20,000 in China and 10,000 to 20,000 in the United States.^[4,5]^ Most of the patients often need by surgery which may include osteotomy, total hip arthroplasty, or core decompression. Since not all cases with steroid therapy develop being steroid-induced ONFH, the prevention of the disorder by risk factors of individual differentiation to steroids sensitivity would be a significant tactic for cases who get steroid therapy.^[6]^

The cytochrome P4503A (CYP3A) is a remarkable enzyme responsible for metabolizing the steroids, and its activities varies more than 10-fold, low CYP3A activity leads to a predominant increase of steroid levels.^[7,8]^ Previous studies have reported the importance of the CYP3A subfamily in the metabolism of statins and that genetic polymorphisms of *CYP3A5* may affect the lipid-lowering responses and pharmacokinetic profiles of statins.^[9,10]^ The *cytochrome P4503A4* (*CYP3A4*) also manifests an approximate 40-fold degree of interindividual polymorphic variation, including *CYP3A1–5* alleles, which have been associated with reduced activity of CYP3A4.^[11]^ The CYP3A4 activity, which metabolizes steroids, was suggested to be associated with the development of osteonecrosis.^[12]^ Meanwhile, Single nucleotide polymorphisms (SNPs) identified in *CYP3A4* rs12333983 (7q22. 1), rs3735451 (7q22. 1), rs2242480 (7q22.1), rs4646437 (7q22.1), and rs2246709 (7q22.1) are associated with the incidence of ONFH in European population or in the animal model.^[12–15]^

This study was aimed at understanding whether the polymorphism of *CYP3A4* gene was associated with a propensity to develop steroid-induced ONFH in the Chinese patients.

## 2 Methods

### 2.1 Ethics statement

The protocol in this study was strictly conformed to the principles expressed in the Declaration of Helsinki and was approved by the Ethical Committee of Zhengzhou Traditional Chinese Medicine Traumatology Hospital. Signed informed consent was obtained from each participant.

### 2.2 Study participants

All analyses were restricted to Chinese Han in our study population. In total, 150 steroid-induced ONFH cases were enrolled in the study from April 2015 to February 2016 in the Zhengzhou Traditional Chinese Medicine Traumatology Hospital in Zhengzhou city, China. These cases had received standard steroid therapy more than 1 year after using >2000 mg of prednisone.^[16]^ The Arlet and Ficat classification was used for radiographic evaluation.^[17]^ Anteroposterior and frog view X-rays of both hips were done in all of the patients. Confirmed the diagnosis of ONFH in patients without X-ray changed by using the magnetic resonance imaging. All steroid-induced ONFH cases had no previous history of prior chemotherapy or radiotherapy. All cases were recently diagnosed and confirmed to get steroid-induced ONFH.

A total of 250 healthy unrelated individuals as the controls from June 2015 to February 2016 were recruited from the medical examination at Zhengzhou Traditional Chinese Medicine Traumatology Hospital. All the participants were restricted to Chinese Han who lived in Zhengzhou city and its surrounding areas. Generally, subjects with chronic diseases and conditions involving vital organs such as brain heart, liver, and lung were excluded from this research. The aim of the above exclusion standards was to minimize the known factors that influence the variation of human complex diseases.

### 2.3 Genotyping

Genomic deoxyribonucleic acid (DNA) was extracted from blood samples using the phenol–chloroform extraction method,^[18]^ and DNA concentration was measured by spectrometry (DU530 UV/VIS spectrophotometer, Beckman Instruments, Fullerton, CA). We used Sequenom MassARRAY Assay Design 3.0 Software (Sequenom Inc., San Diego, CA) to design Multiplexed SNP MassEXTEND assay.^[19]^ SNP genotyping was performed using the Sequenom MassARRAY RS1000 (Sequenom Inc., San Diego, CA) with standard protocol recommended by the manufacturer.^[19]^ Data management and analyses were performed using Sequenom Typer 4.0 software (Sequenom Inc., San Diego, CA) as previously described.^[19,20]^

### 2.4 Statistical analysis

The genotype frequencies of each SNP in the control subjects were checked using the Hardy–Weinberg equilibrium (HWE). Data analysis was performed using SPSS version 16.0 statistical package (SPSS, Chicago, IL) and Microsoft Excel (Chicago, IL). The significance of the difference of alleles and genotype frequencies between the groups was tested using the chi-square method.^[21]^*P* < 0.05 was considered to represent statistical significance. Differences in the distribution were analyzed using logistic regression. Odds ratios (ORs) and 95% confidence intervals (CIs) were tested using unconditional logistic regression analysis with adjustment for age and gender.^[22]^ The 3 genetic models (Dominant, Recessive, and Additive) were applied by PLINK software (Chicago, IL) (http://pngu.mgh.harvard.edu/purcell/plink/) to assess the association of single SNPs with the risk of steroid-induced ONFH. The analyses of linkage disequilibrium (LD), and haplotype construction was used by the Haploview software package (version 4.2) (Chicago, IL).^[23]^

## 3 Results

The analysis included 150 cases (89 males, 61 females; mean age 41.96 ± 11.49 years) who were receiving steroid treatment and 250 healthy controls (151 males, 99 females; mean age 43.27 ± 9.64 years). The basic characteristics of patients and control subjects are illustrated in Table 1. As listed in Table 2, a multiplexed SNP MassEXTEND assay was designed with the Sequenom MassARRAY Assay Design 3.0 Software. Chromosomal, position, band, HWE *P* value, alleles A/B, minor allele frequency (MAF) control, and MAF case for 5 SNPs are shown in Table 3. Meanwhile, we found that rs2242480 in the *CYP3A4* gene was associated with steroid-induced ONFH as a risk factor in the allele model (*P* = 0.023; OR: 1.47; 95% CI: 1.05–2.04). All of the tested SNPs are in HWE in the control population of this study.

**Table 1:**
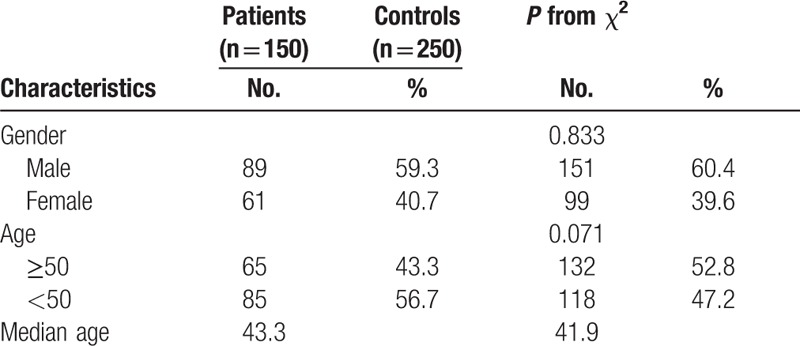
Basic characteristics of recruited individuals.

**Table 2:**
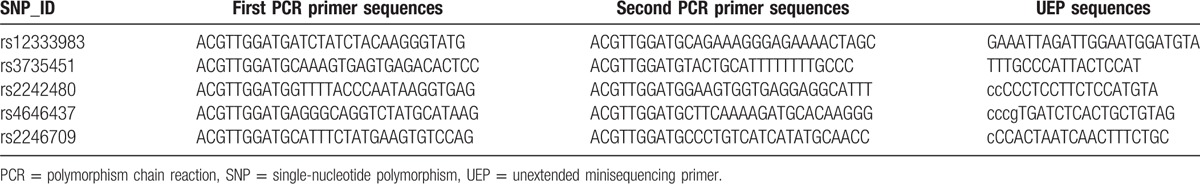
PCR primers in this study.

**Table 3:**
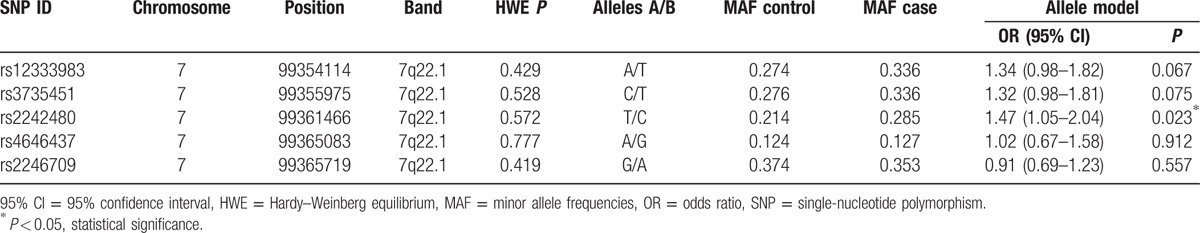
Examined SNPs examined in the *CYP3A4* gene.

Association results between *CYP3A4* SNP genotypes and the risk of steroid-induced ONFH are listed in Table 4. The significant associations were observed between the genotype “T/C” of rs2242480 and increased steroid-induced ONFH risk in the additive model (crude *P* = 0.027; OR: 1.44; 95% CI: 1.04–1.99; adjusted by age + gender *P* = 0.028; OR: 1.44; 95% CI: 1.04–1.99). The genotype “T/C” of rs2242480 was found to be associated with increased risk of steroid-induced ONFH (crude *P* = 0.049, OR, 1.51; 95% CI: 1.00–2.27).

**Table 4:**
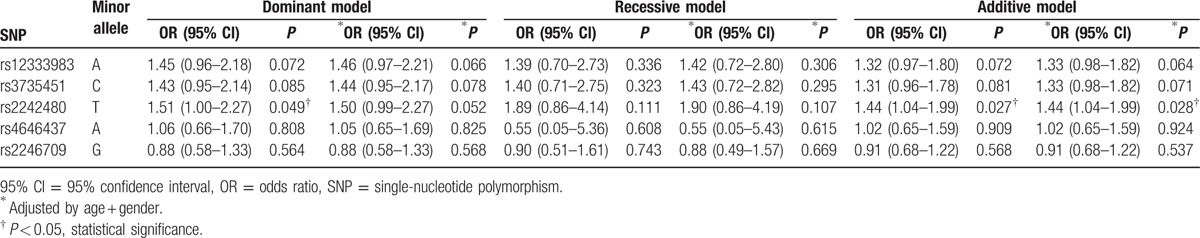
Association between single-nucleotide polymorphism genotypes and risk of steroid-induced ONFH.

**Figure 1:**
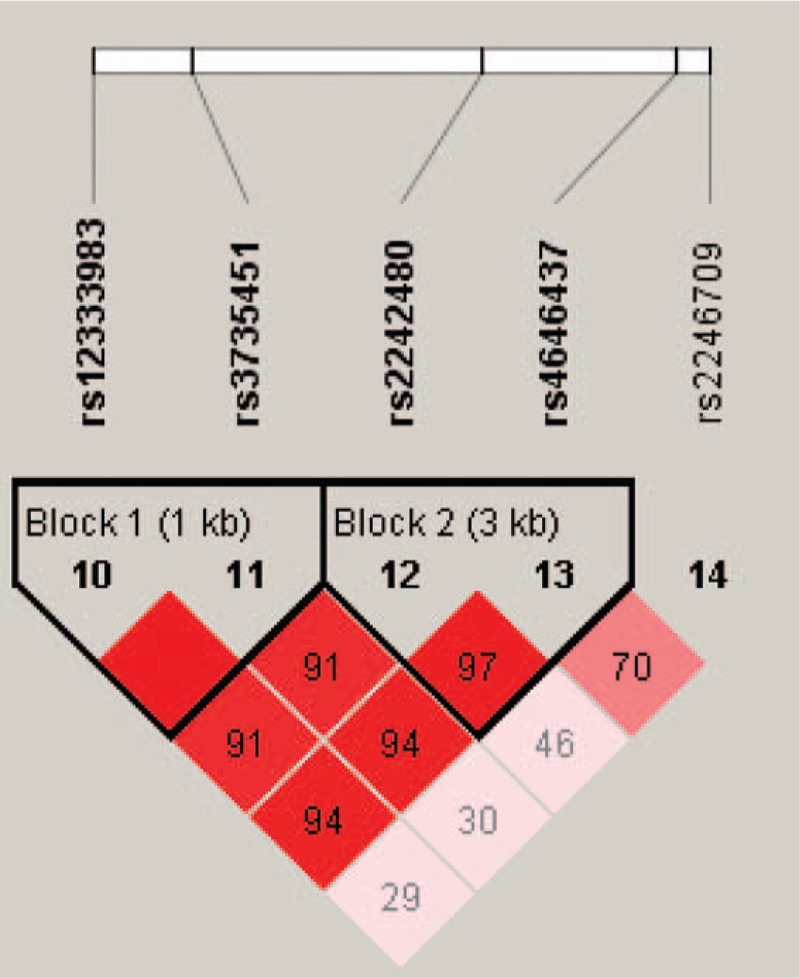
Haplotype block map for 5 single nucleotide polymorphisms (SNPs) of the *cytochrome P4503A4* gene. Block 1 includes rs12333983 and rs3735451; Block 2 includes rs2242480 and rs4646437. The linkage disequilibrium between 2 SNPs is red schemes.

Two blocks (rs12333983 and rs3735451 in the Block1; rs2242480 and rs4646437 in the Block 2) were detected in studied *CYP3A4* SNPs by haplotype analyses (Fig. 1). The results of the association between the *CYP3A4* haplotype and the risk of steroid-induced ONFH are listed in Table 5. Haplotype “TG” in Block 2 was found to be associated with a risk factor of steroid-induced ONFH (crude *P* = 0.005, OR, 1.81; 95% CI: 1.18–2.76; adjusted by age + gender *P* = 0.006; OR: 1.81; 95% CI: 1.18–2.76). Haplotype “CG” in Block 2 was found to be associated with a protective factor of steroid-induced ONFH (crude *P* = 0.024, OR, 0.68; 95% CI: 0.49–0.95; adjusted by age + gender *P* = 0.025; OR: 0.69; 95% CI: 0.49–0.95).

**Table 5:**
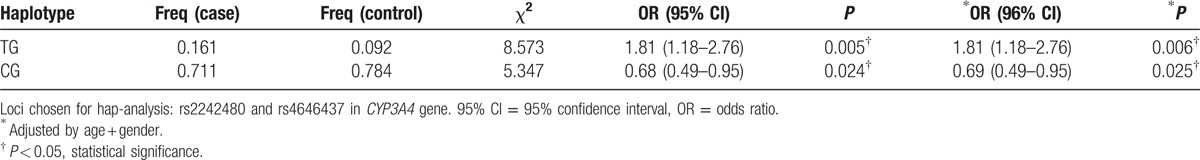
*CYP3A4* haplotype frequencies associated with the risk of steroid-induced ONFH.

## 4 Discussion

In our case–control study, we identified rs2242480 in the *CYP3A4* gene associated with an increased risk of steroid-induced ONFH in the allele model and the additive model adjusted by age + gender. A protective effect was observed for the haplotype “TG” of the *CYP3A4* gene that was associated with decreased risk of developing steroid-induced ONFH. In addition, we also observed a strong effect of the “CG” haplotype, which increased the risk of developing steroid-induced ONFH.

Our results indicate a statistically significant difference between the steroid-induced ONFH and control groups regarding the *CYP3A4* SNPs, suggesting a positive association between genetic polymorphism and the susceptibility of steroid-induced ONFH. In the previous study, Kitada et al^[24]^ showed the product of the *cytochrome P450* gene, *CYP3A4*, is considered to be the main cytochrome responsible for steroid metabolism. The cytochrome P450 family is a group of enzymes included in the oxidative and reductive metabolism of almost all lipid-soluble medicines. However, one of the potential causes of nontraumatic ONFH, lipid metabolism abnormality, is occlusion of vessels responsible for blood supply of the femoral head.^[25]^ This might root in exposure of the femur to lower methylpredonisolone concentration for shorter duration of time by intensive metabolism in the liver by enhanced CYP3A activity. Although the exact mechanism to do harm to the osteal circulation by the high level and prolonged exposure to the exogenous steroid keep up to be researched, the CYP3A activity played an essential role that no doubt brought about development of steroid-induced ONFH.^[26,27]^

In the study, we found that genotype “T/C” of rs2242480 in intron 10 of the *CYP3A4* gene, which is mapped to chromosome 7q22.1, was associated with the risk of steroid-induced ONFH in Chinese Han patients. The most frequent mutant allele of rs2242480 in the *CYP3A4* was characterized by a G to A substitution at position 82266. The allele frequency of rs2242480 was 0.22 to 0.37 in the Chinese population.^[28,29]^ There is an evidence of association between the mutant of rs2242480 and clopidogrel response variability in Spanish patients with coronary artery–related disease.^[30]^ In addition, this mutant genotype was associated with a higher CYP3A metabolic activity, thus enhancing the formation of metabolites, specifically for prodrugs, which are activated via CYP3A through biotransformation.^[31]^ According to previous researches, polymorphisms of *CYP3A4* gene associated with steroid-induced ONFH had been proved in the Japanese population. It was reported that it was an allele that appeared with high frequency in Japanese and Oriental people. It is considered that the genetic polymorphism of *CYP3A4* was an important cause of individual differences in drug metabolism in Oriental people. These results were consistent with the results of our present study.^[12,32]^ Thus, the exact location and biological functions of the real causal SNPs in the *CYP3A4* gene is of great interest and warrants further investigation.

Haplotype analysis suggested that steroid-induced ONFH risk was substantially elevated among individuals with specific haplotypes. In the previous study, Li et al^[13]^ found the association between *CYP3A4* rs4646437 T > C and tacrolimus pharmacokinetics. Because the *rs2242480* and *rs4646437* genes are both located in 7q21.1, the LD between rs2242480 and rs4646437 might influence the affect of *CYP3A4* SNPs on the tacrolimus concentration (*C*_0_/*D*). Crettol et al^[33]^ reported that the rs4646437-T allele was in strong LD (*r*^2^ = 0.82) with the rs2242480-G allele in Caucasian renal transplant recipients. Chau et al^[34]^ found that genetic polymorphisms of the *CYP3A4* gene included in serum concentrations were significantly correlated with finasteride metabolism. The homozygous minor allele of rs2242480 and rs4646437 in *CYP3A4* were associated with lower finasteride levels. Block 2 included 2 SNPs (rs2242480 and rs4646437), and we found the haplotype association analyses showed that haplotype “TG” was associated with the increased risk of steroid-induced ONFH crude/adjusted by age + gender. Interestingly, haplotype “CG” was found to be associated with a decreased risk of steroid-induced ONFH crude/adjusted by age + gender. This explanation of results need to do the further researches.

There are several limitations to our study. First, the sample size (150 patients and 250 control subjects) was not relatively large among steroid-induced ONFH association studies. Second, steroid-induced ONFH patients and healthy controls were used in the same hospital to avoid selection bias. As we all know, population admixture was confounding factor and can caused type-I errors (false positive) in association analysis. Third, we also performed Bonferroni correction analysis and found no statistical significant associations between *CYP3A4* SNPs and steroid-induced ONFH risk. However, this may be due to the weakness of Bonferroni correction itself. True important differences may be considered nonsignificant, and the likelihood of type II errors are also elevated.^[35]^ We also performed a power analysis that showed that the power of rs2242480 was 0.76 and it was >0.75.

In conclusion, our comprehensive analysis of SNPs in the *CYP3A4* gene indicates that *CYP3A4* genotypes and haplotypes are associated with steroid-induced ONFH risk in Chinese Han population. In the further researches, if SNP is evaluated as a risk marker, filtrating patients at high risk of ONFH would be possible, steroid dosage could be basic on individual differences and it could prevent the development of steroid-induced ONFH.
